# Data Leakage in Health Outcomes Prediction With Machine Learning. Comment on “Prediction of Incident Hypertension Within the Next Year: Prospective Study Using Statewide Electronic Health Records and Machine Learning”

**DOI:** 10.2196/10969

**Published:** 2021-02-11

**Authors:** Alexandre Chiavegatto Filho, André Filipe De Moraes Batista, Hellen Geremias dos Santos

**Affiliations:** 1 Department of Epidemiology School of Public Health University of São Paulo São Paulo Brazil

**Keywords:** machine learning, data leakage, prediction

Applications of machine learning algorithms to predict the incidence of health outcomes have an enormous potential to improve clinical practice and lower health care costs [1]. Machine learning is a subset of artificial intelligence that uses data to improve decisions through experience, which is especially promising in a data-driven world. Dr Ye and colleagues’ article on hypertension incidence prediction in the *Journal of Medical Internet Research* adds to this literature [2], but its potential contribution and applicability are hindered by a major flaw.

The objective of the study was to “develop and validate prospectively a risk prediction model of incident essential hypertension within the following year.” The authors follow good prediction protocols by applying a high-performing machine learning algorithm (XGBoost) and by validating the results on unseen data from the following year. The algorithm attained a very high area under the curve (AUC) value of 0.870 for incidence prediction of hypertension in the following year.

The authors follow this impressive result by commenting on some of the most important predictive variables, such as demographic features, diagnosed chronic diseases, and mental illness. The ranking of the variables that were most important for the predictive performance of hypertension is included in a multimedia appendix; however, the above-mentioned variables are not listed near the top. Of the six most important variables, five were: lisinopril, hydrochlorothiazide, enalapril maleate, amlodipine besylate, and losartan potassium. All of these are popular antihypertensive drugs.

Data leakage occurs when one or more features used to train the algorithm has hidden within itself the result of the outcome, and is considered one of the most frequent mistakes in machine learning [3]. This is different from predictive importance, that is, the relative effect of each variable in increasing or decreasing the expected outcome, as it usually comes after the outcome. Therefore, it is a consequence of the outcome that is being predicted and not the other way around.

A classic example from machine learning textbooks is the inclusion of the ID number of the patient as a predictor. While this should not have predictive importance if randomly assigned, it is common that patients coming from the same hospital have similar ID numbers in multicenter data sets. In the case of cancer prediction, for example, machine learning algorithms will learn that similar ID numbers that come from oncology hospitals have a higher probability of cancer.

As an example, we used real data to test the effect of including mechanical ventilation to predict intensive care unit (ICU) admission among patients with COVID-19 [4]. This is another example of data leakage, as mechanical ventilation usually only occurs after ICU admission and should not be used to predict its risk. Figure 1 shows the decrease in the prediction metrics for ICU admission with the exclusion of mechanical ventilation as a predictor, with the area under the ROC (receiver operating characteristic) curve decreasing from 0.76 to 0.64, and precision from 0.49 to 0.17.

**Figure 1:**
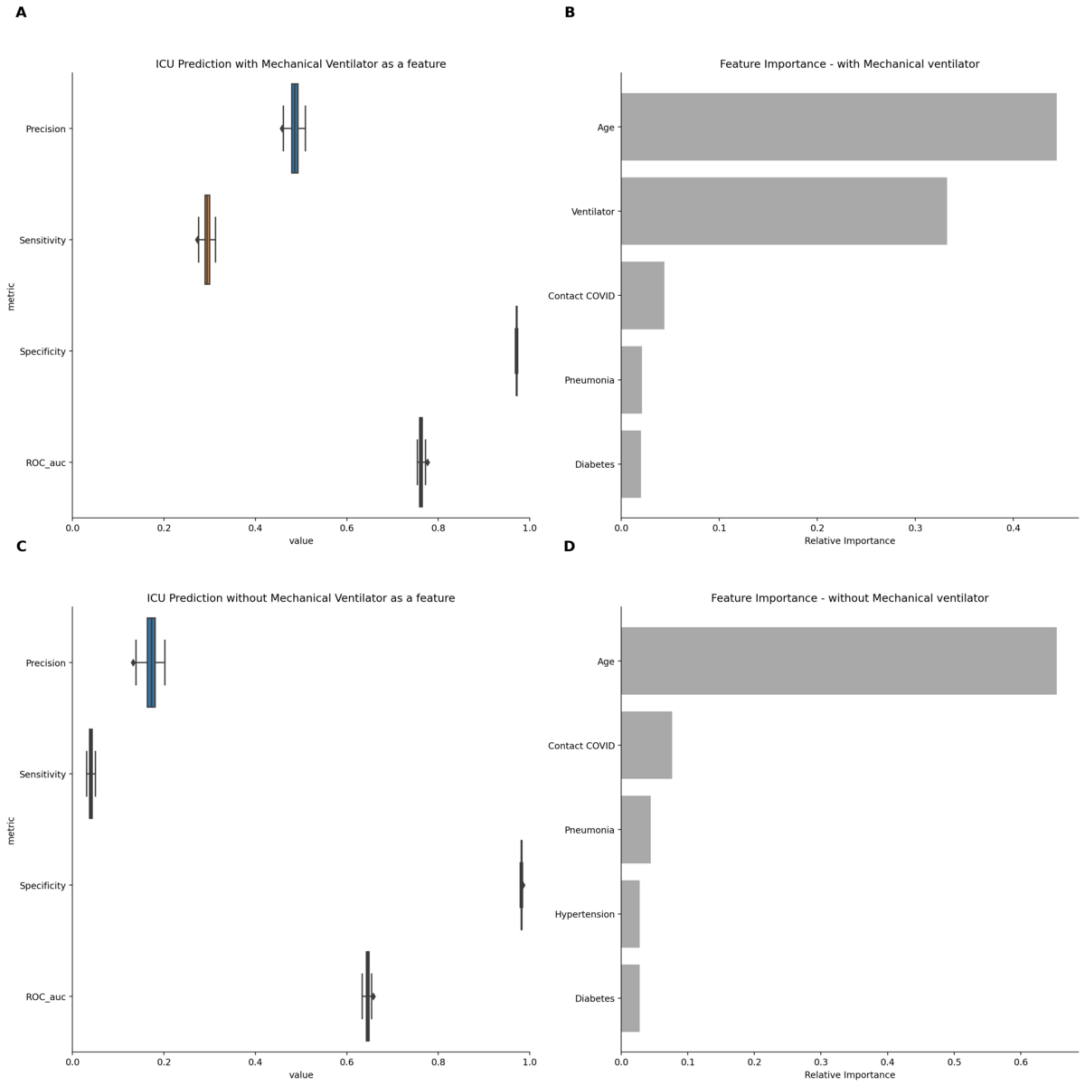
Performance metrics for the prediction of intensive care unit (ICU) admission with and without the use of mechanical ventilation as a predictor.

By including the use of antihypertensive drugs as predictors for hypertension incidence in the following year, Dr Ye and colleagues’ work opens the possibility that the machine learning algorithm will focus on predicting those already with hypertension but did not have this information on their medical record at baseline. While this would work for a prediction competition, where data science teams compete to produce the best predictive model such as in a Kaggle challenge [5], it is not of particular scientific or clinical interest. In the case of the latter, just one variable (the use of a hypertension drug) is sufficient for physicians to infer the presence of hypertension, while for the former, the knowledge of this being a highly predictable event (as measured by the AUC) is severely impaired.

In order to identify the presence of data leakage in prediction studies, it is important to have a conceptual pathway of how the predictors longitudinally affect the outcome variable, as there is no statistical method that is capable of pointing out the presence of data leakage. Improving the predictive performance of specific data sets for different diseases is an important new field in epidemiology and data science. The authors can still contribute to this literature by providing the new AUC of the prediction after addressing the data leakage issue.

## Abbreviations

AUC: area under the curve
ICU: intensive care unit
ROC: receiver operating characteristic
